# The association between the amount of fetal fraction in cell-free DNA testing and adverse pregnancy outcomes: A cohort study

**DOI:** 10.18502/ijrm.v22i11.17824

**Published:** 2025-01-10

**Authors:** Maryam Aryavand, Maryam Nurzadeh, Marjan Ghaemi, Sina Eskandari Delfan, Vajiheh Marsoosi

**Affiliations:** ^1^Department of Obstetrics and Gynecology, Shariati Hospital, Tehran University of Medical Sciences, Tehran, Iran.; ^2^Vali-E-Asr Reproductive Health Research Center, Family Health Research Institute, Tehran University of Medical Sciences, Tehran, Iran.

**Keywords:** Noninvasive prenatal testing, Cell-free DNA, Pre-eclampsia.

## Abstract

**Background:**

Noninvasive perinatal testing is a new method of screening for aneuploidy called cell-free DNA (cfDNA). Fetal fraction (FF) plays a crucial role in assessing the reliability of aneuploidy detection through noninvasive perinatal testing.

**Objective:**

We aimed to investigate the association between the amount of FF in cfDNA testing and adverse pregnancy outcomes.

**Materials and Methods:**

This cohort study was conducted on 619 singleton pregnant women who were candidates for cfDNA testing and were referred to the perinatology clinics of Shariati hospital and Arash Women's hospital, both affiliated with Tehran University of Medical Sciences, Tehran, Iran from March 2019 to June 2020. The FF was extracted from the cfDNA test results, and the participants were followed until delivery.

**Results:**

A total of 619 singleton pregnant women with a mean ± SD age and FF of 34.4 ± 4.85 and 8.39 ± 3.95, respectively, participated in the study. A significant association between maternal age and FF was not found (p = 0.12). A lower FF was associated with a rise in the incidence of gestational diabetes mellitus (p = 0.02) and a higher FF was associated with a rise in the incidence of fetal growth restriction (p < 0.001). However, high or low FF was not associated with pre-eclampsia, premature rupture of membranes, birth weight, or delivery time. No significant association was found between FF and multiple of the median of pregnancy-associated plasma protein-A and free β-human chorionic gonadotropin.

**Conclusion:**

The amount of FF may be considered a predictor of certain adverse pregnancy outcomes. Therefore, maternity care should be performed more carefully for women with high or low FF.

## 1. Introduction

The emergence of noninvasive perinatal testing (NIPT) has recently brought about a new method of screening for aneuploidy called cell-free DNA (cfDNA) (1). It is derived from fetal trophoblasts and has a smaller fragment size than maternal DNA. The prenatal cfDNA test has become extensively utilized in clinical settings as a prenatal screening test for common aneuploidies. In comparison to traditional maternal serum screening methods, the cfDNA test has higher sensitivity and specificity in detecting trisomy 21, 18, and 13 (2). cfDNA is highly recommended for individuals aged over 35 yr, those with positive aneuploidy screening with increased nuchal translucency, abnormal ultrasound findings, and positive personal or familial history of aneuploidy (3). Fetal DNA in maternal circulation comes primarily from the placenta and fetal blood cells. During pregnancy, there is an increase in the amount of cfDNA present in the maternal circulation. This increase, referred to as the fetal fraction (FF), ranges from approximately 3–13%. FF, known as the fetal cfDNA to total cfDNA ratio in circulation, plays a crucial role in assessing the reliability of aneuploidy detection through NIPT. Traditional markers found in maternal serum are utilized for assessing aneuploidy risk and predicting adverse pregnancy outcomes (4).

Various technical factors, such as the handling of samples and the selection of bioinformatics tools, can affect FF and its estimation. Several biological factors have been recognized that can impact FF, such as gestational age, weight and/or body mass index (BMI), trisomy, fetal crown-rump length, serum pregnancy-associated plasma protein-A (PAPP-A), serum-free β-human chorionic gonadotropin (β-hCG), hypertension, twins, smoking, and assisted conception, fetal sex, fetal karyotype, and FF is associated with a higher risk of disorders related to the placenta and adverse perinatal outcomes (5).

The percentage of FF increases significantly as the gestational age progresses and there is a significant negative correlation between FF and the maternal BMI and age (6). An association exists between low FF in prenatal cfDNA testing and several diseases including pregnancy-induced hypertension and pre-eclampsia after 34 wk, gestational diabetes mellitus (GDM), aneuploidy, and congenital structural anomalies (7). Mothers who have a high FF during the first trimester of pregnancy are at elevated risk of delivering a small for gestational age infant < 5 ${\;}^{\text{th}}$ percentile (8). Several studies have shown low FF in cfDNA testing is associated with adverse pregnancy outcomes (4, 7, 9–12).

## 2. Materials and Methods

### Study setting

This cohort study was carried out on 619 singleton pregnant women aged between 17 and 51 yr referred to the perinatology clinics of Shariati hospital and Arash Women's hospital, both affiliated with Tehran University of Medical Sciences, Tehran, Iran. The study period spanned from March 2019 to June 2020. The pregnant women had undergone a cfDNA test or had been requested to undergo one during this period. To examine cfDNA and determine FF, maternal blood samples were collected and sent to referral laboratories for analysis. The FF was extracted from the cfDNA test results, and the participants were followed until delivery.

### Eligibility criteria 

Pregnant women were included in the study if they had a singleton pregnancy and had undergone NIPT. Women who have had multiple pregnancies, BMI ≥ 35 kg/m², medical diseases such as lupus, diabetes, hypertension, or if they had undergone an elective termination of pregnancy before 37 wk, and FF < 4% (a minimum of 4% FF cutoff is necessary to obtain informative results), were excluded. Additionally, women who were candidates for termination of pregnancy due to aneuploidy, cases of cfDNA-positive aneuploidy, and cases of severe pre-eclampsia such as hemolysis, elevated liver enzymes, and low platelet count syndrome, as well as eclampsia, and multiorgan involvement were excluded from the study.

### Data collection and outcome measurements

The cfDNA test was conducted at a gestational age of 10–15 wk. Questionnaires and supplementary information were completed at the beginning of the study and participants were followed during pregnancy and until delivery. The gestational age of all pregnant women was determined according to the last menstrual period and based on the crown-rump length, as determined by ultrasound.

In this study, spontaneous abortion, fetal growth restriction (FGR), pre-eclampsia, gestational diabetes, rupture of membranes, preterm labor (PTL), and also the results of the screening tests conducted during the first trimester were considered adverse pregnancy outcomes. Preterm birth refers to a spontaneous delivery that occurs before the completion of 37 wk of pregnancy. Labor pain was characterized as the presence of regular contractions of at least 4 contractions within 30 min and progressive changes of the cervix (dilatation ≥ 1 cm or effacement ≥ 50%) when the pregnant woman was under observation. The cases that terminated at 37 wk due to premature rupture of membranes (PROM) or spontaneous delivery before 37 wk were included in the PTL group. Cases terminated at 37 wk of pregnancy due to mild pre-eclampsia were included in the delivery group after 37 wk. The study did not include other cases of elective termination of pregnancy before 37 wk of pregnancy.

FGR was characterized as having an abdominal circumference or estimated fetal weight that fell below the 3 ${\;}^{\text{rd}}$ percentile, or as having an abdominal circumference or estimated fetal weight below the 10 ${\;}^{\text{th}}$ percentile and elevated umbilical artery pulsatility index above the 95 ${\;}^{\text{th}}$ percentile. GDM was defined as fasting blood sugar ≥ 92 mg/dl, 1 hr oral glucose tolerance test ≥ 180 mg/dl, and 2 hr oral glucose tolerance test ≥ 153 mg/dl at 24–28 wk of pregnancy with 75 gr of glucose, and in the subsequent follow-ups, they needed a diet or drug treatment for blood sugar control. Pre-eclampsia was characterized as systolic blood pressure ≥ 140 mmHg or diastolic blood pressure ≥ 90 mmHg, which occurs for the first time after 20 wk of pregnancy (at least 2 measurements taken at least 4 hr apart) along with proteinuria of ≥ 300 mg/24 hr or proteinuria (+1) in a urine sample taken at random. Cases of severe pre-eclampsia such as hemolysis, elevated liver enzymes, and low platelet count syndrome, eclampsia, and multiorgan involvement were not included in the study.

10 cc of peripheral blood from each mother was collected in ethylenediaminetetraacetic acid tubes for analysis. The semiconductor sequencing platform method was used to investigate aneuploidy disorders based on cell-free fetal DNA, which includes plasma separation, DNA extraction, automated library construction, automated polymerase chain reaction (library amplification and enrichment), DNA library sequencing, and automated data analysis. The sample preparation and testing were conducted using the ion torrent platform and premarital kits. The FF was extracted from the result of the cfDNA test and PAPP-A and free β-hCG as multiple of median from first-trimester screening tests. Fetal tests including cfDNA, pregnancy tests, and sonography were performed, and a checklist was completed based on the results of history-taking and medical records.

To examine cfDNA and determine FF, maternal blood samples were collected and sent to referral laboratories for analysis. Once the cfDNA was isolated, it was sequenced using high-throughput sequencing technology that generated millions of short DNA sequences, which were then analyzed bioinformatically to determine the FF. The FF was calculated by comparing the quantity of sequencing reads that originated from the fetal genome to the total number of sequencing reads. This was typically reported as a percentage or fraction.

### Sample size

G power software was used to estimate the sample size and examine differences between the mean FF in the 2 FGR and normal groups. With an average of 8% in the normal group and an average of 11% in the FGR group, along with a standard deviation (SD) of 5% in each group and alpha set at 0.05 and beta at 0.2, a sample size of 619 people was obtained.

### Ethical Considerations

This study was approved by the Ethics Committee of the Tehran University of Medical Sciences, Tehran, Iran (Code: IR.TUMS.MEDICINE.REC.1399.571). Informed consent was obtained from all participants before enrolment, and their information remained confidential.

### Statistical Analysis

Quantitative variables were presented as mean and SD, and qualitative variables were reported as percentages. To compare quantitative variables, the *t* test or ANOVA was utilized, while the Chi-square test was utilized for qualitative variables. A p-value < 0.05 was deemed statistically significant. The statistical analysis was performed using SPSS version 21.

## 3. Results

619 singleton pregnant women were analyzed. The mean ± SD of FF of these pregnant women was 8.39 ± 3.95. A significant inverse association was observed between FF and maternal BMI (p = 0.01 and Pearson correlation coefficient = -0.248). The basic characteristics of the participants are shown in table I.

The mean ± SD of FF in women with FGR was 10.24 ± 4.9 and in women without FGR was 8.29 ± 3.8. A significant difference was observed in FF between the 2 groups (p < 0.001). No significant association was observed between FF and delivery time. The mean ± SD of FF was 7.16 ± 3.5 in women with GDM and 8.54 ± 3.95 in women without GDM. A significant difference in FF was observed between the 2 groups (p = 0.02). FF in women with pre-eclampsia was not significantly different from those without this disorder. FF in women with PROM was not significantly different from those without this disorder. A comparison of adverse pregnancy outcomes based on the participants FF is shown in table II.

The mean ± SD of the weight of the infants was 3059.8 ± 705.51 and no meaningful correlation was observed between FF and the weight of the infants (p = 0.10). Among the participants, 71 women had completed the first-trimester screening tests. The mean ± SD of PAPP-A and β-hCG were 1.14 ± 0.93 and 1.69 ± 1.25, respectively and no meaningful correlation was observed between FF and PAPP-A (p = 0.17) and between FF and β-hCG (p = 0.12).

**Table 1:** Basic characteristics of the participants

	**Participants (n = 619)**	
**Variables**	**Mean ± SD**	**P-value**
	**Age (yr)**	34.4 ± 4.85	0.12
**Weight (kg)**	68.77 ± 11	0.14
**Height (cm)**	163 ± 7.04	0.08
**BMI (kg/m²)**	25.6 ± 5.2	0.01
*t* test. BMI: Body mass index		

**Table 2:** Comparison of adverse pregnancy outcomes based on the FF of the participants

**Variables**		Contribution*	**Median**	FF**	**IQR**	**P-value**	**95% Confidence Interval **	
**Termination**								
	**Abortion (termination < 20 wk)**	38 (6.1)	7	8.7 ± 4.3	4	0.154	6.4684	11.0316
	**Delivery (between 20–37 wk)**	146 (23.6)	8/5	8.9 ± 4.6	6/75	0.123	8.1901	9.6866
	**Term (delivery after 37 wk)**	435 (70)	8	8.2 ± 3.7	5	0.104	7.8611	8.5665
**FGR**		33 (5.5)	10	10.24 ± 4.9	8/5	< 0.001	0.56245	3.34141
**PROM**		76 (12.3)	9	9.3 ± 4.0	4/75	0.070	-2.06376	-0.17969
**GDM**		92 (15.2)	10	7.16 ± 3.5	6	0.020	0.51859	2.25260
**PE**		49 (8.2)	8	8.3 ± 3.6	5	0.931	-1.17840	1.07866
*Data presented as n (%). **Data presented as Mean ± SD, ANOVA, and *t* test. FF: Fetal fraction, IQR: Interquartile range, FGR: Fetal growth restriction, PROM: Premature rupture of membrane, GDM: Gestational diabetes mellitus, PE: Pre-eclampsia								

## 4. Discussion 

The significance of the correlation between FF and adverse pregnancy outcomes lies in the potential clinical implications for prenatal care and management (13). Our results showed low FF was associated with GDM and high FF was associated with FGR, while high or low FF was not associated with pre-eclampsia, PROM, infant weight, gestational age, PTL, spontaneous abortion, preterm birth, PAPP-A, and free β-hCG. A significant inverse association was seen between FF and BMI.

### FF and PTL

No significant association was observed between PTL and FF. Our findings do not concur with 2 prior studies (12, 14) which demonstrated that FF may be a predictor of PTL. One of these studies (12) was conducted on 370 mothers with a gestational age of 16–17 wk. They investigated pregnancy complications in 2 groups: mothers with low FF and mothers with normal FF. PTL was higher in the group of mothers with low FF.

### FF and spontaneous abortion

In the present study and another research (12), no significant correlation was found between FF and pregnancy loss before the 20 ${\;}^{\text{th}}$ wk of pregnancy, but a previous study showed that cfDNA testing can be employed to identify chromosomal anomalies in miscarriages where the FF is sufficiently elevated (15).

### FF and FGR

High FF may be a predictor of the occurrence of FGR. Our findings are inconsistent with the results of a previous study indicating that low FF in cfDNA testing correlates with impaired fetal growth due to placental dysfunction (9).

### FF and infant weight

A previous study found that there was no discernible correlation between low Apgar scores, small for gestational age, large for gestational age, low birth weight, macrosomia, and low FF concerning neonatal outcomes (16). These results are not consistent with our findings. In our study, no association was found between FF and infant weight. This result may be a predictor of the high predictive value of FF for fetal defects in the future.

### FF and pre-eclampsia

In this study, no meaningful association was observed between FF and pre-eclampsia. In one study, blood pressure disorders related to pregnancy were observed in people with low FF in the 16–17 ${\;}^{\text{th}}$ wk of pregnancy (12), and in another study, it was reported that the precise timing of cfDNA elevation in pre-eclampsia is not clear, but it starts to rise before the onset of clinical symptoms, reaching a high level approximately 3 wk before the onset of clinical symptoms of pre-eclampsia (17). The results of one prior study showed that low FF of cfDNA in asymptomatic women may indicate a potential risk for future hypertensive disease and placental dysfunction (11). Another cohort study was conducted on 2191 singleton mothers who underwent NIPT during 13–26 wk of gestation, and it was found that low FF is associated with the occurrence of pre-eclampsia in singleton pregnancies (4). A study also found that low FF in cfDNA testing was associated with pregnancy-related hypertensive disorders (9). A retrospective cohort study was conducted on 2063 mothers, and it was found that the incidence of pregnancy-induced hypertensive diseases was significantly higher in low FF cases (16). These results were not consistent with our findings.

### FF and GDM

Low FF may be a predictor of the occurrence of GDM. This result concurs with the results of 2 prior studies, which indicated that lower FF was associated with an increased risk of certain pregnancy complications, such as GDM (12, 18).

### FF and PAPP-A and free β-hCG

Our results, in contrast to one study, showed that the presence of FF in maternal plasma cfDNA increased with PAPP-A and β-hCG (19).

### FF and BMI

Our study showed a significant inverse association between FF and BMI. 2 other studies showed FF in maternal plasma cfDNA decreases as maternal BMI increases (19, 20). These results corroborate our findings.

### FF and preterm birth

Our findings are consistent with the results of a previous study (11) that demonstrated that no correlation was found between low FF and the occurrence of preterm birth, but was not consistent with other research (9) that revealed low FF in cfDNA testing is correlated with the occurrence of preterm birth. The observed divergence may be attributed to variations in study methodologies, populations, methods of genome sequencing, methods of aneuploidy detection, and gestational weeks at which the blood samples were collected.

While this study provides valuable insights into the potential association between FF and adverse pregnancy outcomes, further studies are required to confirm these findings. Consequently, it is imperative to interpret the results with caution and to consider the broader context of the existing literature on prenatal screening and diagnosis. The strength of our multicentric study lies in investigating the diagnostic role of FF in a wide spectrum of adverse pregnancy outcomes. However, our study has limitations, particularly concerning using different laboratories for analysis. For future investigations, we recommend using a unique referral laboratory for more accurate analysis.

High or low FF may serve as a predictor of adverse pregnancy outcomes. In the event of either high or low FF, the maternity care provided should be conducted with greater caution. FF is not typically reported in many laboratories. It is recommended that all laboratories perform the FF test and identify FF as a noninvasive prenatal screening.

##  Data Availability

Data supporting the findings of this study are available upon reasonable request from the corresponding author.

##  Author Contributions

All authors had full access to all of the data in the study and takes responsibility for the integrity and accuracy of the data analysis. Concept and design: V. Marsoosi. Acquisition, analysis, or interpretation of data: M. Aryavand, M. Nurzadeh, V. Marsoosi, and S. Eskandari Delfan. Drafting of the manuscript: S. Eskandari Delfan. Critical revision of the manuscript for important intellectual content: All authors. Statistical analysis: M. Aryavand. Supervision: M. Nurzadeh and M. Ghaemi.

##  Acknowledgments

This article has been extracted from M.D. Thesis by Maryam Aryavand. The authors would like to express their gratitude to all participants who assisted with the implementation of this project. Artificial intelligence was not used in any way in the current study.

##  Conflict of Interest

The authors declare that there is no conflict of interest.
